# Passive Fluidic Chip Composed of Integrated Vertical Capillary Tubes Developed for On-Site SPR Immunoassay Analysis Targeting Real Samples

**DOI:** 10.3390/s120607095

**Published:** 2012-05-29

**Authors:** Tsutomu Horiuchi, Toru Miura, Yuzuru Iwasaki, Michiko Seyama, Suzuyo Inoue, Jun-ichi Takahashi, Tsuneyuki Haga, Emi Tamechika

**Affiliations:** NTT Microsystem Integration Laboratories, Atsugi 240198, Japan; E-Mails: miura.toru@lab.ntt.co.jp (T.M.); iwasaki.yuzuru@lab.ntt.co.jp (Y.I.); seyama.michiko@lab.ntt.co.jp (M.S.); inoue.suzuyo@lab.ntt.co.jp (S.I.); takahashi.junichi@lab.ntt.co.jp (J.T.); haga.tsuneyuki@lab.ntt.co.jp (T.H.); tamechika.emi@lab.ntt.co.jp (E.T.)

**Keywords:** SPR, immunoassay, raw sample, on-site, passive pump, microfluidics

## Abstract

We have successfully developed a surface plasmon resonance (SPR) measurement system for the on-site immunoassay of real samples. The system is composed of a portable SPR instrument (290 mm(W) × 160 mm(D) × 120 mm(H)) and a microfluidic immunoassay chip (16 mm(W) × 16 mm(D) × 4 mm(H)) that needs no external pump system. An integrated vertical capillary tube functions as a large volume (150 μL) passive pump and a waste reservoir that has sufficient capacity for several refill operations. An immunoassay was carried out that employed the direct injection of a buffer and a test sample in sequence into a microfluidic chip that included 9 antibody bands and 10 reference reagent bands immobilized in the flow channel. By subtracting a reliable averaged reference sensorgram from the antibody, we effectively reduced the influence of the non-specific binding, and then our chip successfully detected the specific binding of spiked IgG in non-homogeneous milk. IgG is a model antigen that is certain not to be present in non-homogeneous milk, and non-homogeneous milk is a model of real sample that includes many interfering foreign substances that induce non-specific binding. The direct injection of a real sample with no pretreatment enabled us to complete the entire immunoassay in several minutes. This ease of operation and short measuring time are acceptable for on-site agricultural, environmental and medical testing.

## 1. Introduction

The on-site immunoassay of real samples is expected in various fields such as medicine, healthcare [1–4], food analysis [5], and environmental analysis [6–9]. A surface plasmon resonance measurement system is often used in immunoassays because of its high sensitivity combined with a simple method [10–19]. We have developed a portable SPR measurement system. The combination of a portable SPR measurement system and a microfluidic device is one way to achieve on-site immunoassays without the complex pretreatment of real samples [20].

The microfluidic device has been applied to immunoassay analysis [21] and will be a powerful tool for the SPR measurement of real samples. Since the sensitivity of SPR measurement is highest at the surface, a flow is effective in reducing confusing signals caused by impurity sedimentation.

However, the use of an external conventional pump system has many disadvantages including a large dead volume, a troublesome tube connection, and the need to wash the pump system after every measurement. These difficulties are fatal for on-site measurement.

Although flow cells incorporating a mechanical micro-pump have been studied using MEMS technology [22–27], these cells appear to be too expensive to apply to immunoassay in the fields of healthcare or food analysis where many tests must be processed. In these fields, devices that come into contact with real samples are expected to be single-use to eliminate the possibility of cross contamination. An inexpensive and mass-producible micro-pump system [28,29] is clearly needed for real sample immunoassays.

A micro-pump system driven by capillary force is the simplest and has been widely studied for many applications [30–35]. However the single capillary is limited in terms of flow volume and flow rate. The velocity of liquid front under its own capillary pressure is inversely proportional to the length already filled with liquid [36].

Many previous studies whose goals were to increase the total flow volume and prevent any reduction in the flow rate were undertaken by making the inside wall of a flow cell geometrically complex to increase the area in contact with the liquid sample [37,38]. These structures, where the cavity has many built-in pillars or many branched micro-trenches, are fabricated using lithographic techniques. The flow volume of flow cells including these structures as passive pumps were limited because of their two dimensional structure. The passive pumps of integrated capillaries, which are formed in the thickness direction of the substrate, are expected to have a large flow volume despite their small footprint.

In this work, we developed an immunoassay chip that includes a passive flow function driven by the capillary force of integrated vertical capillaries and demonstrated an SPR immunoassay using model antigen-spiked non-homogenized milk as an example of a real sample. Our final goal is to achieve on-site immunoassay at milking stations to make it possible to detect antigens related to fast-spreading infectious diseases. The approach would help minimize economic damage to dairy farms by facilitating quick decisions regarding the suitable treatment or isolation of affected cows. For this purpose we must prepare a ready-to-use measurement chip that enables the on-site assay of a raw milk sample to be completed in several minutes, which reflects the typical total time needed to milk a cow. In this paper, we investigated the detection performance of our sensor chip using a model antigen (human IgG) that did not exist in the raw milk we used, which included many kinds of foreign substances without any sample pre-treatment.

## 2. Experimental

### 2.1. Instrument, Materials and Reagents

**Figure 1.**
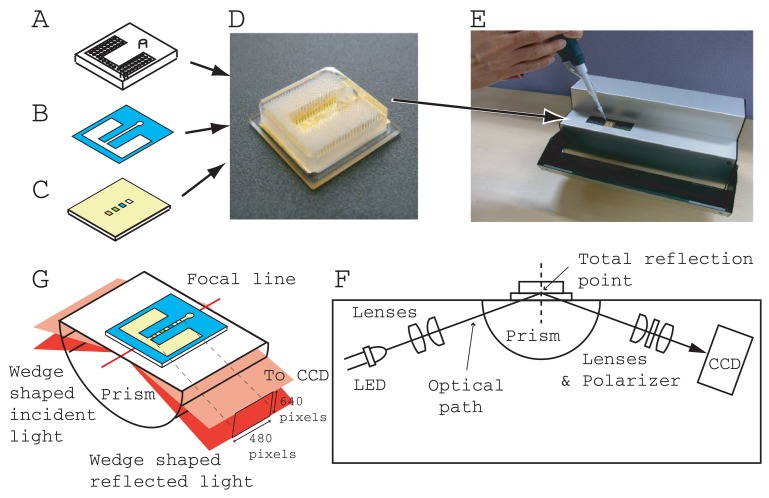
Structure of microfluidic chip for SPR measurement composed of integrated vertical capillary enclosure made of clear acrylic resin (**A**), thin plastic flow channel film (**B**), gold/titanium sputtered substrate with immobilized antibodies and enzyme in band array configuration (**C**), and photograph of finished product (**D**). The chip is mounted on a portable SPR instrument and sample liquid is injected with pipette (**E**). Schematic diagram of the portable SPR instrument, (**F**). Optical configuration at the interface of the instrument and the sensor chip, (**G**).

A portable SPR instrument (290 mm(W) 160 mm(D) × 120 mm(H)), (Smart SPR SS-1001, NTT Advanced Technology, Japan) (Figure 1(E)) and a homegrown control and data acquisition program coded with LabVIEW (National Instrument, Japan) was used for measurement. The SPR instrument had a Kretschmann type optical configuration (Figure 1(F)). The sensing area (4.5 mm × 0.3 mm) is located on a center of focused line of a cylindrical prism (BK7, 1.51 refractive index). The incident light was 770 nm wavelength from LED. A CCD (480 × 640 pixels) camera detected the reflection intensity with a resolution of 4.5 mm/480 pixels along the sensing line and a reflection angle of 10 degrees/640 pixels (Figure 1(G)). The CCD output was transferred to a laptop PC (ThinkPad T7500, Lenovo Japan, Japan) through a gigabit Ethernet cable with a 20 millisecond interval and was averaged every second. We thus obtained the reflection intensity minimum, SPR angle, by employing the ordinary centroid curve fitting program.

Clear and black acrylic resin plates were purchased from Mitsubishi Rayon Co. Ltd., (Japan), and Sumitomo Chemical Co. Ltd., (Japan), respectively. Double-sided adhesive films (TL470S 75 μm thickness) and (PTT25, 50 μm thickness), were supplied by Lintec Corporation, (Japan) and Kimoto Co. Ltd. (Japan), respectively.

Human IgG (I4506), anti human IgG (I3382), and anti protein A (P3775) were obtained from Sigma Aldrich, Co., (MO, USA). Rabbit anti SPEC (Streptococcal Pyrogenic Exotoxin C) (R5V153-754) and sheep anti Staphylococcal alpha Hemolysin (S5V156-754) were purchased from Meridian Life Science, (ME, USA). Rabbit polyclonal to Streptococcal Pyrogenic Exotoxin B (anti SPEB) (ab53403) and mouse monoclonal to streptolysin (ab23501) were purchased from Abcam plc. (UK). Mouse anti SED (Staphylococcus aureus Enterotoxin D) (BM1327) was purchased from Acris Antibodies GmbH, (Germany). Bovine prothrombin (enzyme) (CP3049U) was purchased from Fitzgerald Industries International (MA, USA). Mouse anti Staphylococcus Enterotoxin B, C2, D (anti SEB), (FU84002206) was purchased from Funakoshi Co., Ltd., (Japan). Blocking reagent (Block Ace) was purchased from DS Pharma Biomedical Co., Ltd. (Japan). Phosphate-buffered saline (PBS) was obtained from Invitrogen Co., (CA, USA). Non-homogenized milk was supplied by Kisuki Nyugyo Co. (Japan). Homogenized milk was supplied by Meiji Nyugyo Co. (Japan).

### 2.2. Chip Design and Fabrication

Figure 1 shows the structure of an immunoassay chip developed for SPR measurement. The chip has three parts, an acrylic resin enclosure (2 or 3 mm thickness) (Figure 1(A)) with integrated capillary tubes and an inlet hole, a thin plastic two-sided adhesive film (0.05 or 0.075 mm thickness) (Figure 1(B)), which is opened along a flow line, and a gold/titanium sputtered optically transparent substrate (1 mm thick) with several kinds of immobilized antibody and enzyme in a band array configuration (Figure 1(C)). The top acrylic resin part was fabricated with a CO_2_ laser cutting-machine (VL 200, Universal Laser Systems Inc, AZ, USA) from a large acrylic resin base plate. The integrated capillary tubes and inlet hole (3 mm diameter) were opened along the thickness direction and the lengths of all the capillary tubes and the inlet hole were the same as the thickness of the base plate. It took a few minutes to complete the enclosure part. The designed radius of the capillary was 0.1 mm. The distance between adjacent capillary centers was 0.36 mm. The number of capillaries and the total capacity of the integrated capillaries are summarized in Table 1. Our fluidic chip was designed for single-use. The vertical capillary tubes function as a passive pump and a waste reservoir. Therefore, there is no outlet on our fluidic chip.

The flow channel was fabricated from double-sided adhesive film and using a mechanical cutting plotter (CG60ST Mimaki Engineering Co., Ltd., Japan). The center flow channel (0.9 mm wide) branched to the left and right compartments (See Figure 1(B)). Both compartments and the branching node were connected with the bottoms of the integrated capillaries. The upstream center flow channel was located at the bottom of inlet.

This special shape of flow channel has an advantage for the beneficial use of the limited footprint of the sensor chip, whose sensing area must be arranged at the center of the chip with top priority.

The biologically sensitive material (ligand, antibody and enzyme) and blocking reagent were diluted with ion-exchanged water and immobilized on the gold surface of the substrate in band array configuration using the spotter (Nano-Plotter 2.1, Gesim mbH, Germany). Each band was 0.2 mm × 0.44 mm in size and was arranged in parallel with the band center distance of 0.25 mm within SPR sensing area. There were 19 bands: 9 ligand bands and 10 reference bands (blocking reagent). The reference bands were arranged on both sides of the ligand bands. After washing the gold surface including the spot area in running water to remove excessive sediment from the gold surface, it was exposed to blocking solution (2% blocking reagent diluted with water) for 20 min to reduce the contribution of the non-specific binding effect. The gold surface was again washed in running water and then air-dried. Blocking the non-specific binding reaction is indispensable in immunoassays in order to reduce background noise. In addition, the use of the flow method in immunoassays is effective in increasing the signal of the specific binding reaction caused by the quick supply of antigens and is effective in reducing the background noise caused by impurity sedimentation in real samples.

The three parts were assembled using a custom-made alignment jig. The chip size was 16 mm (W) × 16 mm (D) × 3 or 4 mm (H). The final product (Figure 1(E)) was packaged in an aluminum sealing bag with a desiccating agent and stored in a refrigerator until the measurement was performed (within two weeks).

### 2.3. Measurement

After mounting the measurement chip on the SPR instrument, the sample solution was injected through an inlet hole with a pipette. The diameter of the inlet hole was 3 mm and it was 2 or 3 mm high, and the pipetting volumes were less than 14 and 21 μL, respectively. Because the total capacity of the integrated capillary was larger than that of the inlet, if the volume of the sample solution was larger than the capacity of the inlet hole, the solution was divided and injected several times according to the requirements of the experiment.

## 3. Results and Discussion

### 3.1. Flow Characteristics

**Figure 2.**
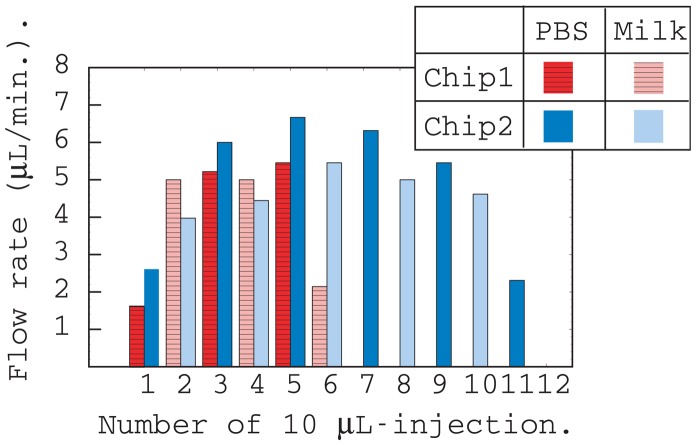
Flow rate distributions of passive fluidic chips calculated from the run-off time of 10 μL injections into the inlet. The run-off time was measured by observing the bottom of the inlet.

Figure 2 shows the results of flow rate measurements of chips 1 and 2 listed in Table 1. The flow rate was obtained by measuring the time taken for 10 μL of liquid to run off from the inlet. PBS and homogenized milk were injected alternately for both chips.

The liquid flow rates were approximately the same for both chips except for the initial and final injections. The flow rate of milk was slower than that of PBS because milk is more viscous than PBS. The total flow volume of chips 1 and 2 were evaluated from the number of injections and were 60 and 110 μL, respectively. These values were approximately equal to the calculated values listed in Table 1, and include the capacity of the thin layer region surrounded by adhesive film.

The driving force of the flow for the fluidic chip is the capillary pressure at the liquid front, which depends on the geometrical configuration. Our fluidic chip is constructed using two basic three-dimensional shapes, namely, a cylinder (inlet hole, micro capillary tube) and a cuboid (center flow channel, flow channel below capillary tubes, namely a junction zone). The capillary pressures at the inlet, *P_inlet_*, the cuboid center channel, *P_cc_*, the junction zone located near the outlet of the center channel, *P_jz_*_cc_, and the micro capillary, *P_mcapi_*, are expressed as below [36,39].

(1) $$P_{\text{inlet}}=-2\gamma \cos\theta \;(1/r_{\text{inlet}})$$

(2) $$P_{cc}=-2\gamma \cos\theta \;(1/d+1/\omega )$$

(3) $$P_{\text{jzcc}}=-2\gamma \cos\theta \;(1/d+1/\omega_{jz})$$

(4) $$P_{\text{mcapi}}=-2\gamma \cos\theta \;(1/r_{\text{mcapi}})$$

where, *d, ω, ω_jz_, r_inlet_* and *r_mcapi_* are the depth and width of the center channel, the width of the junction zone, and the radii of the inlet and the micro capillary, respectively. *γ* is the surface tension. Here, for simplicity, all the contact angles were set at the same value, *θ*

The relationships between the capillary pressures of our fluidic chip were designed using the geometrical parameters (*d, ω, ω_jz_, r_inlet_* and *r_mcapi_*), to satisfy the inequality expression below,

(5) $$P_{cc}<P_{\text{jzcc}}<P_{\text{mcapi}}<P_{\text{inlet}}$$

Even if the refill operations were repeated many times, the center channel was always full of fluid, because the absolute value of the capillary pressure at the center channel *P_cc_* was the largest. The ability to perform repeated refill operations without drying is an advantage for the immunoassay of a desiccated chip as described later.

### 3.2. Ligand Patterning

**Figure 3.**
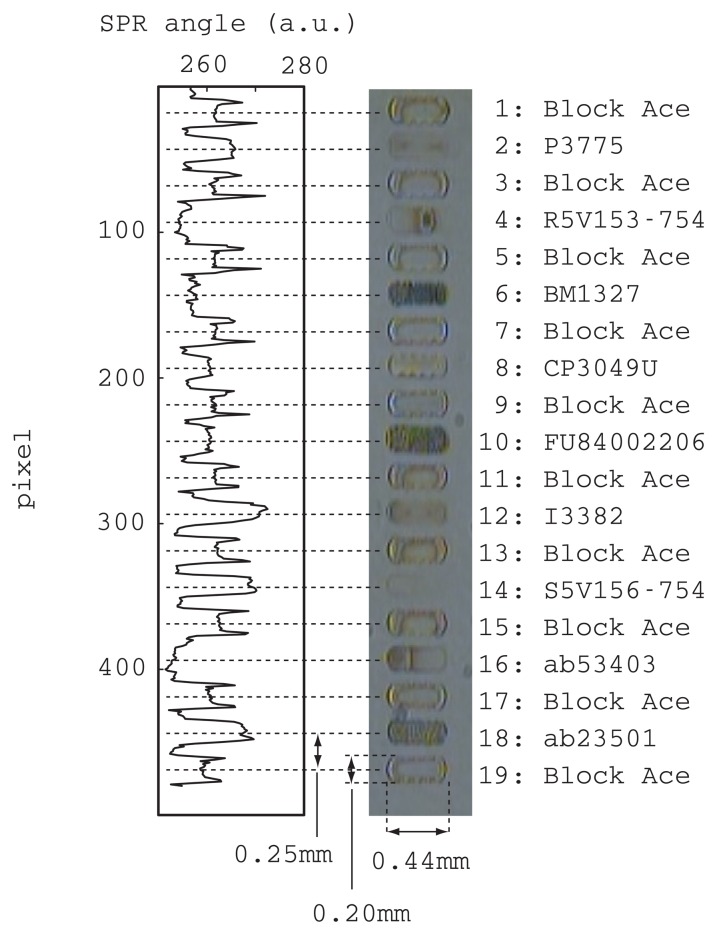
Photograph of spotted antibodies and enzyme in a band array structure (**center**), their abbreviations (**right**) and the SPR signal at the center of each band (**left**). The blocking reagent (Block Ace) was spotted on both sides of the antibodies and enzyme as a reference in difference sensorgrams.

Figure 3 shows a photograph of spotted antibodies, an enzyme and a blocking reagent. The abbreviations for these materials are listed to the right of the photograph with a reference number that is used in the following discussions. All the spots had a band configuration that was realized by connecting a few droplets in a line. The bandwidth, length, and distance between band centers were, 0.20, 0.44 and 0.25 mm, respectively. All the bands were arranged in parallel without any crossing. The liquid will flow above these bands in an orthogonal direction, which is from the top to the bottom of the picture.

This photograph was taken after all bands had been dried. The odd numbered bands were all a blocking reagent and they were used as references in the SPR measurements discussed later. The spot patterns of the blocking reagent were all similar and clear edges can be seen. On the other hand, the antibodies and enzyme had individual patterns. Although bands 2 and 14 were unclear, they were certainly spotted as evidenced by the SPR signal shown on the left side of the picture. This SPR signal was measured with the center channel filled with blocking reagent. The vertical axis corresponds to the position along the center flow channel and the horizontal axis corresponds to the refractive index.

After spotting, the substrate surface was washed with running water and exposed to blocking reagent. At this point, all the band images of the ligands and blocking reagents disappeared from the microscope view. However in the SPR view, clear trapezoids could be seen at exactly the position of spotting except for bands 4, 6 and 16. Some possibilities were considered for this disappearance from the SPR signal. Either these antibodies were completely removed during the post process of spotting, or these antibodies had the same refractive indexes and adsorbed the same amount as the blocking reagent employed in the post process. Further experiments are needed to overcome this problem. Nevertheless, we were able to confirm that a sufficient amount of ligands was immobilized on the substrate for our further experiment.

### 3.3. SPR Sensorgram

**Figure 4.**
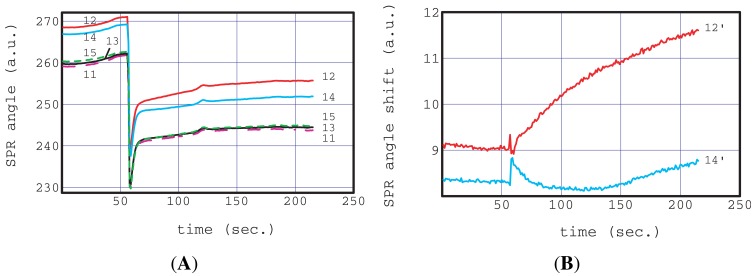
Immunoassay analysis of spiked IgG in non-homogenized milk using a portable SPR instrument and a microfluidic chip. The selected raw sensorgrams A and their difference sensorgrams B. The selected sensorgrams are measured at the specific antibody of the target antigen: anti human IgG (12:I3382), the non-specific antibody of the target antigen: anti Staphylococcal alpha Hemolysin (14:S5V156-754), and their references on both sides. Each difference sensorgram is obtained by subtracting an average reference sensorgram from that of the antibody. An average reference sensorgram is the average of reference sensorgrams located on either side of the antigen.

Figure 4 is one example of the result of an SPR immunoassay of 10 μg/mL human IgG (I4506) in non-homogenized milk. In all the following experiments, black chips (chip 3) were used to reduce the affect of stray light caused by fluctuations in the outside light intensity during the pipetting operation above the chip.

SPR sensorgrams were obtained by selecting CCD pixels from 480 pixels by adjusting to the region of interest. There were 19 bands in the chip and sensorgrams were obtained at the center position of each band as a representative value. This is because the center position of each band seems to be stable and reliable as can be seen in left graph of Figure 3. For example, all the blocking reagent bands have rabbit ears with different heights.

Figure 4(A) shows the raw response of bands 11 to 15. The flow operation of a SPR sensorgram of a real sample (non-homogenized milk) was two-step injected into the inlet hole using a pipette. The first injection was 4 μL of 10% of blocking reagent. The second injection was an actual sample, IgG spiked non-homogenized milk. The first injection of 4 μL is too small to produce sufficient flow. This blocking reagent solution filled and remained in the center channel and a partial region of the junction zone. This state is very stable because the absolute value of the capillary pressure at the center channel is the largest in our chip as described above (Equation (5)). We waited 300 s in this state and then injected the IgG spiked non-homogenized milk. This waiting time was considered enough for the activation of the dried antibodies, enzyme and blocking reagent.

The SPR instrument started recording 60 s before the milk injection. All the sensorgrams in Figure 4(A) show large steps at 60 s, caused by the refractive index change of bulk solution from the blocking reagent to the IgG spiked non-homogenized milk. The observation of these large steps indicates that the interfacial boundary of the two liquids was clear in the sensing region. This provides excellent conditions for the estimation of the analyte concentration for comparison with the adsorption curve based on concentration change according to the step function. The sensorgrams of the blocking reagent band (11, 13, 15) have almost the same profile over the entire time region.

On the other hand, those of the antibody (12:I3382, 14:S5V156-754) bands have similar profiles to the blocking reagent band but their absolute values were different. The similarity of the sensorgrams of blocking reagent bands means that these bands are suitable for reference use. The differences between the absolute values of these antigens were reflected in their original characteristics, namely, the amount of immobilized antibodies and their refractive indexes.

A small profile difference can be seen between sensorgrams 12 and 14 within 60 s of the milk injection. This difference can be clearly seen in Figure 4(B) in the reference subtracted sensorgrams, 12′ and 14′. 12′ is the result of the subtraction of the average of 11 and 13 from 12, and 14′ is that of the subtraction of the average of 13 and 15 from 14.

The shape of sensorgram 12′ is a typical adsorption curve based on an antigen-antibody (I4506-I3382) reaction. All other antibody and enzyme bands were similar to 14′ (not shown in the figure) and to no adsorption curves.

A small unexpected peak was observed at 120 s in each sensorgram as shown in Figure 4(A). The origin of the small peak appears to be a large particle approaching the antibodies and blocking reagent bands. However this unexpected signal was successfully removed from the subtracted sensorgrams. This good ability to remove nonessential signals caused by, for example, non-specific adsorption, approaching particles and bubbles, is because reliable and homogeneous references were arranged plurally very close to the antibody of interest.

From these results, we conclude that our chip and measurement protocol successfully detects antigen-antibody reactions by isolating them from non-specific adsorption in an analyte solution that includes a number of different foreign substances with no pretreatment.

### 3.4. Calibration

A total of 29 chips (chip 3) were examined to investigate the antigen concentration dependence of the SPR signal. 4, 4, 3 and 18 chips were measured for 10, 5, 1 and 0 μg/mL of human IgG (I4506) in non-homogenized milk. In the SPR measurement, the slope of the sensorgram is proportional to the analyte concentration. We evaluate the slope by linear fitting using sensorgram obtained 150 s after milk injection.

**Figure 5.**
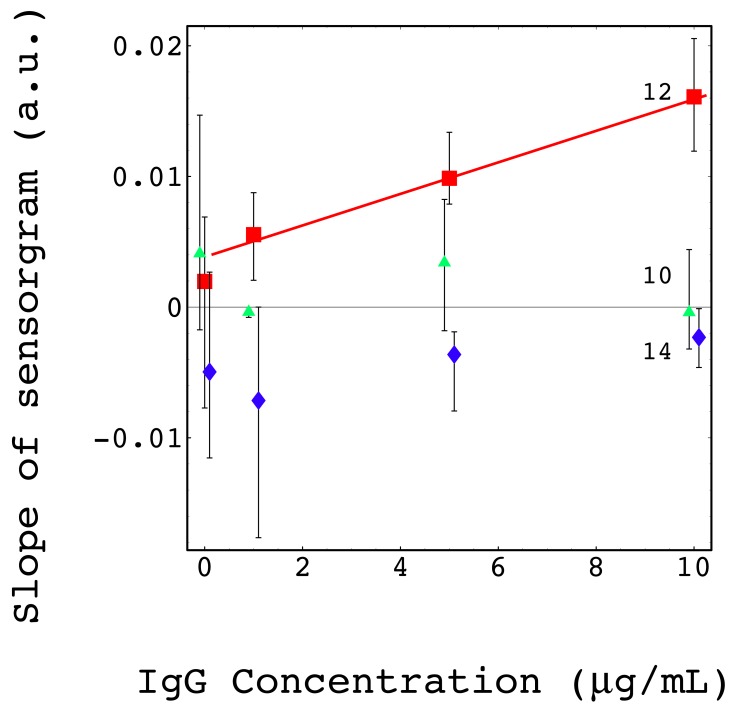
Calibration curve of the SPR immunoassay measured with a spiked antigen in non-homogenized milk. The relationship between the antigen concentration and the slope of the sensorgrams. Specific antigen-antibody pair 12 (squares) and non-specific pairs 10 (triangles) and 14 (diamonds) are shown in the same graph. A small offset in the horizontal axis was used for non-specific pairs to give clear view of the error bar overlaps. The solid line in the graph is the result of linear regression analysis applied to the specific pair.

Figure 5 shows the relationship between the IgG concentration and slope of the SPR sensorgrams. The legend numbers correspond to the antibody numbers in Figure 3.

The values of antibodies 10 and 14 respectively were plotted with −0.1 and 0.1 μg/mL offsets to see the error bars without an overlap. A good linear relationship with a positive slope was obtained only with antibody 12 (I4506-I3382 reaction). Other antibodies and enzyme showed no clear positive slopes as found with antibodies 10 and 14 (not shown in Figure 5).

The linear regression analysis method was applied to all antibodies and enzyme. Table 2 summarize the coefficient of determination R squared and adjusted R squared to judge the goodness of the linear fitting.

Both R squared and adjusted R squared of antibody 12 (I3382) were about 0.7, which is much larger than that of any other antibodies and enzyme. This result confirms that only antibody 12 (I3382) could detect antigen I4506 with reasonable concentration dependence. The fitting function derived from the linear regression was

(6) y=0.00229441+0.00141756x

The noise level was defined as the average value at 0 μg/mL, 0.001966, then the detection limit of 1 μg/mL has an S/N ratio of 2.8.

## 4. Conclusions

We have developed an SPR measurement system (portable instrument and microfluidic immunoassay chip) for the on-site immunoassay of real samples. The microfluidic immunoassay chip includes a capillary pump consisting of integrated vertical capillary tubes, a thin layer flow channel and a base plate immobilized band array of antibodies and enzyme. The capillary pump can allow liquid to flow at an almost constant flow rate and when we execute several refill operations by employing manual pipetting. The bands of blocking reagent positioned on both sides of the antibodies and enzyme were excellent references for removing the non-specific adsorption of foreign substances in real samples. Although the sensitivity should be improved, the developed system could perform an immunoassay on a few μL of a real sample in several minutes without any pretreatment.

This system has many advantages for on-site immunoassays. The immunoassay chip was designed for single use to prevent cross contamination and the design employed a pipetting operation. Pipetting is the simplest and most fundamental method for sampling real samples and transferring them to other places. The waste products for one measurement are a pipette chip and an immunoassay chip. There is no need for tube or syringe cleaning and so no cleaning solution is needed unlike in ordinary flow analysis. Even if the test sample includes bio-hazardous material, safe operation can be expected because the test sample liquid is keep inside the chip after measurement.

It is necessary to mass-produce immunoassay chips at low cost for use in testing many samples. The main parts of this chip were made from inexpensive polymer, and the fabrication methods were also low cost. We were able to achieve a combination of inexpensive material and a low cost fabrication method because tight sealing is not necessary for this chip since the capillary force exerts negative pressure.

The microfluidic components were quickly and easily fabricated and the microfluidic design can be changed easily to meet a user's request. The choice of biologically sensitive material (antibody, enzyme, DNA, aptamer) and its combination allow us to expand the application range. The chip has the possibility to be used as a prototype before mass production.

We expect our immunoassay system consisting of direct injection without a pretreatment sample to be widely applied in the fields of medicine, healthcare, food analysis and drug discovery.

## Figures and Tables

**Table 1.** Geometric parameters of integrated capillary tubes and total flow volumes. The designed radius and center distance were 0.1 and 0.36 mm, respectively. The flow volume is the calculated value.

Radius(mm)	Number	Height(mm)	Film Thickness(μm)	Flow Volume(μL)	Note
0.12	468	2	50	47.8	chip1 clear
0.12	870	3	50	123.5	chip2 clear
0.136	816	3	75	150.5	chip3 black

**Table 2.** Coefficient of determination in linear regression analysis method applied to all antibodies and enzyme denoted by numbers in Figure 3.

No.	R Squared	Adjusted R Squared
2	0.011185	−0.025438
4	0.000034	−0.037002
6	0.011498	−0.025113
8	0.005666	−0.031161
10	0.101359	0.068076
12	0.685615	0.673971
14	0.050986	0.015837
16	0.063662	0.028983
18	0.039281	0.003699
